# Qualitative methodological pathway for developing the *Instructive for the Obesity Management in the SUS*, 2018-2020

**DOI:** 10.1590/S2237-96222025v34e20240365.en

**Published:** 2025-04-14

**Authors:** Thanise Sabrina Souza Santos, Larissa Morelli Ferraz Guimarães, Patrícia Pinheiro de Freitas, Suellen Fabiane Campos, Clareci Silva Cardoso, Natacha Toral, Aline Cristine Souza Lopes

**Affiliations:** 1 Universidade de São Paulo, Núcleo de Pesquisas Epidemiológicas em Nutrição e Saúde, São Paulo, SP, Brazil; 2 Universidade Federal de Minas Gerais, Departamento de Nutrição, Belo Horizonte, MG, Brazil; 3 Universidade Federal dos Vales do Jequitinhonha e Mucuri, Departamento de Nutrição, Diamantina, MG, Brazil; 4 Prefeitura Municipal de Belo Horizonte, Secretaria Municipal de Saúde de Belo Horizonte, Belo Horizonte, MG, Brazil; 5 Universidade Federal de São João Del-Rei, Programa Pós-Graduação em Ciências da Saúde, Divinópolis, MG, Brazil; 6 Universidade de Brasília, Departamento de Nutrição, Brasília, DF, Brazil

**Keywords:** Obesity Management, Healthcare Personnel, Continuing Education, Unified Health System, Validation Study, Manejo de la Obesidad, Profesionales de la Salud, Educación Continua, Sistema Único de Salud, Estudio de Validación

## Abstract

**Objective:**

To assess the methodological pathway for developing and validating Instructive for the Obesity Management in the Unified Health System (Sistema Único de Saúde - SUS), focusing on food and nutrition actions, based on the adaptation of the *Dietary Guidelines for the Brazilian Population*.

**Methods:**

The development of the *Instructive of Collective Approach for the Obesity Management in the SUS* included an evaluation of the proposal summary during an in-person expert workshop, and the analysis of the first draft in an in-person validation workshop. In 2018 and 2019, a dynamic carousel was employed during the workshops to record the perceptions of experts from management, academy, and SUS health services. The materials produced during the workshops underwent exploratory content analysis by two authors independently, using theoretical frameworks and theories as predefined categories. Observed divergences were discussed until consensus was reached.

**Results:**

The first workshop, attended by 21 experts, was essential for the decision to develop a material specifically for collective approaches and for defining the theoretical frameworks and theories to be included in the Instructive. The second workshop, with 17 experts, contributed to resolving conceptual issues, addressing discussions and uncertainties, and consolidating of the care flow. The published version of the Instructive incorporated a problem-posing methodology transversally and the transtheoretical model as the core theoretical framework to enhance treatment effectiveness and adherence.

**Conclusion:**

The development and validation of the Instructive followed a scientific research trajectory, contributing to the creation of instructional materials derived from the Dietary Guidelines.

## Resumen

**Objetivo:**

Evaluar el proceso metodológico de desarrollo y validación de una Guía para el Manejo de la Obesidad en el Sistema Único de Salud (SUS), con un enfoque en acciones de alimentación y nutrición, basada en la adaptación de la *Guía Alimentaria para la Población Brasileña*.

**Métodos:**

El desarrollo de la Guía de Enfoque Colectivo para el Manejo de la Obesidad en el SUS incluyó la evaluación de una propuesta de resumen en un taller presencial con expertos y el análisis de la primera versión en un taller presencial de validación. Durante 2018 y 2019, se implementó la dinámica de carrusel en los talleres para registrar las percepciones de los expertos vinculados a la gestión, el ámbito académico y los servicios de salud del SUS. Los materiales generados en los talleres fueron analizados mediante análisis de contenido exploratorio por dos autoras de forma independiente, utilizando marcos teóricos y teorías como categorías predefinidas. Las divergencias observadas fueron discutidas hasta alcanzar consenso.

**Resultados:**

El primer taller, que contó con la participación de 21 expertos, fue clave para la decisión de desarrollar un material exclusivo para el enfoque colectivo y para definir los marcos teóricos y teorías que se incluirían en la Guía. El segundo taller, con la participación de 17 expertos, contribuyó a resolver conceptos, abordar discusiones y dudas, y consolidar el flujo de atención. La versión final publicada de la Guía incorporó transversalmente la metodología problematizadora y utilizó el modelo transteórico como marco teórico principal para potenciar la efectividad y la adherencia al tratamiento.

**Conclusión:**

El desarrollo y la validación de la Guía siguieron un enfoque de investigación científica, contribuyendo a la producción de materiales instructivos derivados de la *Guía Alimentaria para la Población Brasileña*.

## Introduction

Obesity is a public health challenge due to its impact on health, quality of life and the economy. Analyses of global trends from 1990 to 2022 have shown an increase in prevalence in most countries (1), with projections indicating that by 2034, 54% of the world population will have obesity (2).

In Brazil, the prevalence of obesity aligns with the global trend of growth, ranging from 12% to 24% between 2006 and 2023, among adults (3), and may reach 30% by 2030 (4). This epidemiological scenario reflects the complex interaction between multiple determinants of obesity (5), with emphasis on changes in dietary patterns, such as the increased consumption of ultra-processed foods (6), which has been associated with obesity (7,8).

In this context, the second edition of the *Dietary Guidelines for the Brazilian Population* was published in 2014, based on a new paradigm in nutrition science, considering foods and their combinations, and the cultural and social dimensions of diet (9). Since its publication, various materials have been published aiming to support the enhancement of actions to promote adequate and healthy eating (10-16) and to improve the effectiveness of obesity management within the Unified Health System (*Sistema Único de Saúde* - SUS), in line with the need for professional training (17).

As one of the outcomes of the Dietary Guidelines, in 2021, the Ministry of Health, in partnership with the university, published the *Instructive for the Obesity Management in the SUS*, aiming to support health professionals in treating people with obesity (18). It introduces the Strategy for Person with Obesity Care in the SUS, a proposal to organize obesity care in primary health care (PHC) and specialized care (SC), and it includes the Educational Activities Workbook, which features protocols for in-person activities (workshops and environmental interventions) and remote activities (19).

Although the Instructive was recently published by the Ministry of Health, its development process has not yet been scientifically disseminated. Sharing the methodological rigor adopted during its development aims to strengthen its use in continuing education and to guide care practices in the SUS, as well as contribute to the production of instructional materials that assist in the management of other chronic conditions. Thus, this article aimed to evaluate the methodological pathway for developing and validating the Instructive, focusing on food and nutrition actions, adapted from the *Dietary Guidelines for the Brazilian Population*.

## Methods

### Background

The construction of the Instructive was carried out in nine stages, between 2018 and 2020 (Table 1). This qualitative study focuses on describing the last four stages, which have not yet been publicized and are considered crucial for producing a resource that fills existing gaps in obesity management guidance and incorporates new care methodologies. The earlier stages have already been disseminated (20-23).

**Table 1:** Description of the stages of development of the Instructive of *Collective Approach for the Obesity Management* in the Unified Health System

Stage	Participants	Method	Expected outcome
1. Systematic review	Organizing team	Systematic literature review	To gather scientific evidence to develop the Instructive
2. Review of theories and tools for obesity management	Organizing team	Pre-selection and discussion of theories and tools applied in the care for people with obesity	To select theories and tools used in primary health care for discussion during the workshop
3. Diagnosis of structure and processes for obesity management in primary healthcare	Organizing team	Assessment of indicators from the national program access and quality improvement in Primary Care	To understand the level of adequacy of primary healthcare for obesity management
4. Research involving primary healthcare and specialized healthcare professionals	Organizing team	Online research	To identify the main barriers to obesity management
5. Proposal for the content of the Instructive	- Organizing team - General Coordination of Food and Nutrition of the Ministry of Health	Collective development	To propose a draft summary of the Instructive for discussion in the workshop
6. Expert workshop	- Organizing team - Experts	Dynamic carousel	To validate the proposal summary
7. Development of the Instructive	- Organizing team - General Coordination of Food and Nutrition of the Ministry of Health	Review of contributions	To develop the first version of the Instructive
8. Validation workshop	- Organizing team - Experts	Dynamic carousel	To validate first version of the Instructive
9. Development of the final version of the Instructive	- Organizing team - General Coordination of Food and Nutrition of the Ministry of Health	Review of contributions	To provide the final version of the Instructive available for publication by the Ministry of Health

### Researchers’ characteristics

The team responsible for the Instructive’s development included professionals in nutrition, education, psychology, and physical education, with experience in management, academy, and healthcare services within the SUS. The process was conducted in collaboration with the General Coordination of Food and Nutrition of the Department of Prevention and Health Promotion under the Primary Healthcare Secretariat of the Ministry of Health.

### Data collection tool: expert workshop

To assess the summary proposed by the organizing team and the Ministry of Health, an expert workshop was conducted in August 2018, in person, for three hours. Twenty-six health professionals from PHC and SC; municipal, state and federal health managers; and researchers specializing in obesity within the SUS were invited. To promote immersion in the theoretical framework, the experts were provided the proposed summary in advance, which incorporated scientific evidence gathered in earlier stages and a compilation of theories and tools identified during Stage 2 (Table 1): cross-cutting approaches (food and nutrition education and nutrition counseling); theoretical frameworks (motivational interviewing, transtheoretical model, cognitive-behavioral therapy, problem-posing methodology, and supported self-care); and tools used in PHC in individual approaches (shared home care, individual shared care, specific individual care and singular therapeutic project), family approaches (Problem, Roles, Affect, Communication, Time, Illness, Copying, Ecology - P.R.A.C.T.I.C.E. / life cycle / ecomap) and collective approaches (therapeutic and motivational groups, and workshops). This compilation of theories and tools was later published as theoretical material to support obesity management in the SUS. (21).

During the expert workshop a dynamic carousel was implemented, in which all participants walked around different stations to discuss the proposal summary and pre-selected theories and tools, accompanied by a member of the organizing team. At each station, the experts evaluated the strengths and weaknesses of each theory or tool and their application in collective care and individual care of people with obesity.

From the perspective of the chronic care model (24), the experts outlined theoretical frameworks and tools for care and the level of health care (PHC or SC), and the type of approach to be used (collective or individual), based on obesity stratification according to body mass index and the presence of comorbidities (24). Finally, the experts provided suggestions for the final summary. Subsequently, the organizing team proposed a consolidated version of the summary, integrating the suggestions from the workshop. This summary guided the development of the first draft of the Instructive.

### Data collection tool: validation workshop

In August 2019, a second workshop was conducted to validate the content of the Instructive. Also held in person, this eight-hour session invited specialists to evaluate the first draft of the material. Participants were provided the draft in advance for review, with observations recorded through an online tool.

During the workshop, the dynamic carousel was repeated to assess the adequacy of the Instructive’ chapters. At each station, impressions were collected regarding the comprehensiveness of the content and indication of excessive or missing content. The experts walked around the predefined chapter-specific stations supported by a member of the organizing team. Additionally, they evaluated the therapeutic proposals, considering their applicability, alignment with the SUS, use of technologies, feasibility, sequencing and coherence of the activities presented. Suggestions for enhancing their effectiveness were also gathered.

The feedback provided during the workshop was compiled by the organizing team and discussed with the General Coordination of Food and Nutrition of the Ministry of Health. Relevant suggestions were incorporated into the material.

### Data processing and analysis

The outputs of the expert and validation workshops were evaluated through exploratory content analysis (25) to identify the specialists’ perceptions and suggestions and the changes that were incorporated into the consolidated summary version of the Instructive. Theories and tools for obesity management were considered as a priori categories in the exploratory content analysis, serving as references to identify perceptions and suggestions. Changes and the retention of content in the summary and the first version of the Instructive were systematically documented.

The exploratory content analysis was performed using online spreadsheets organized and shared between two authors to enable independent evaluations of perceptions and suggestions. Any observed discrepancies were discussed until a consensus was reached.

For result presentation, the specialists’ perceptions and suggestions were grouped into posteriori categories based on content similarity.

## Results

**Figure 1:**
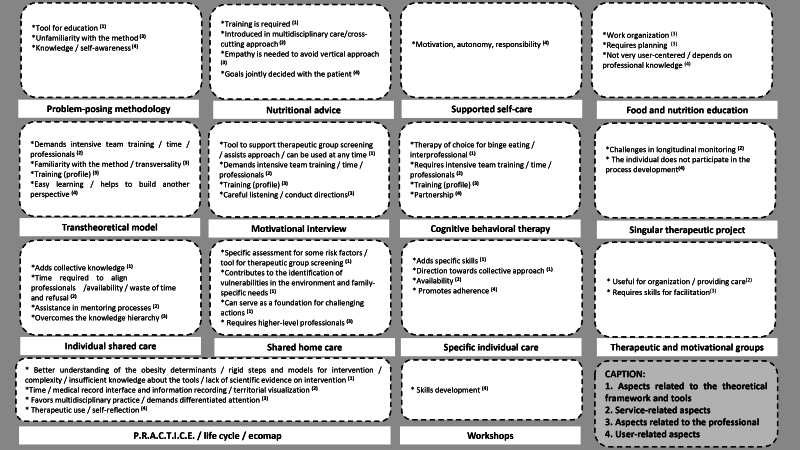
Experts’ perceptions distributed according to the relationship with the theoretical framework or tool, service, professional and user

The expert workshop was attended by 21 specialists, whose perceptions are summarized in Table 2. Perceptions were identified regarding the theoretical framework and the tool (applicability in comprehensive obesity care, enabling a better understanding of its determinants, but requiring qualification); the health service (need to include qualification and mentoring processes into the routine practice, expand the professional team, recognize the territory); the professional (need for training to use the method and collaborative interprofessional practice); and the user (promotion of adherence, self-awareness and skill development) (Figure 1).

**Table 2:** Summary of experts’ perceptions on each cross-cutting approach and theoretical framework

Cross-cutting approaches	
Nutritional advice	- Cross-cutting and multi-professional practice, where the user participates in defining goals with the health team - Requires professional empathy to avoid verticalized practices
Food and nutrition education	- Useful for helping the team organize the work process - Risk of being insufficiently user-centered if professionals are not well-versed in the approach
Problem-posing methodology	- A robust health education tool, with the potential to enhance user self-awareness and engagement in care planning - Unknown to many professionals
Supported self-care	- Promotes user motivation and autonomy - Requires health professionals to tailor its use to the user profile - Demands the availability of supportive materials within health services to ensure its effective application by the team
Theoretical frameworks	
Transtheoretical model	- Staff training is necessary, despite its current application in health services - Can be used by any professional category for screening therapeutic groups - Demands significant time, including more professionals
Motivational interviewing	- Team training is needed, alongside a specific professional profile for its application - Demands significant time, including more professionals - Suitable for screening therapeutic groups, such as those for obesity management - Enables qualified listening and provides appropriate direction for care plans
Cognitive-behavioral therapy	- Allows for interprofessional care - Used in protocols for chronic conditions - Requires significant time and team training - May involve a participation of a psychologist
Singular therapeutic project	- Used in managing more complex cases - Limitations: challenges in longitudinal monitoring and the lack of user participation in the development of the care plan
Theoretical frameworks	
Individual shared care	- Allows for the integration of collective knowledge, and is strategic for mentoring processes and overcoming hierarchical knowledge barriers - Requires time and coordination to align professionals
Specific individual care	- Promotes adherence to treatment - Provides an opportunity to address sensitive issues with greater death, adding specialized skills to care - May face challenges in integrating with the collective approach
Shared home care	- Identifies household vulnerabilities and specific family dynamics - Enables collaboration between community health agents and higher-education professionals
P.R.A.C.T.I.C.E^a^, life cycle and ecomap	- Requires time, considering that the integration with the electronic medical record is incipient and there is standardization for incorporating the tools into the work process - Lack of scientific evidence to support widespread recommendation - Potential to enhance understanding of obesity determinants, recognize diverse family configurations, and facilitate the creation of singular therapeutic projects
Therapeutic and motivational groups	- Useful for organizing care and bringing together users who shared challenges, encouraging the exchange of experiences - Requires health professionals to possess management and facilitation skills - User participation is highly dependent on motivation levels and acknowledgment of obesity as a health issue
Workshops	- The planning and implementation process can affect efficacy - Enable professionals to develop therapeutic and motivational skills - Need for professional training so that the team can maximize the potential of workshops

From the discussion about the tools and their applicability in daily work, the experts recommended the development of Instructive focused on the collective approach and another on the individual approach. Thus, among the theoretical frameworks and theories analyzed in the expert workshop, the following were omitted in the final summary: nutrition counseling, singular therapeutic project, individual shared care, shared home care, specific individual care, and P.R.A.C.T.I.C.E. / life cycle / ecomap.

Regarding the care flow, the experts suggested that the definition of the collective approach should consider the readiness for weight reduction, the severity of obesity and the presence of comorbidities, especially diabetes. To achieve this, the initial assessment should include identifying the stages of change, the degree of obesity and existing comorbidities, adopting the transtheoretical model and cognitive-behavioral therapy, based on the problem-posing approach, as theoretical frameworks for guiding person-centered care and defining therapeutic groups. Thus, the care flow, in the first version of the Instructive, should begin with the stages of change and self-efficacy, constructs of the transtheoretical model, and consider the availability to participate in groups and stratification of the health condition, including the indication for surgical treatment of obesity. The flow included therapeutic groups for: A) People in the stages of pre -contemplation, contemplation and preparation with low self-efficacy; and/or without readiness for group participation (motivational group); B) People in the preparation stage with high self-efficacy, action or maintenance stages without indication for surgical treatment (therapeutic group 1); with indication for surgical treatment (therapeutic group 2); and in post-surgical follow-up or with success in therapeutic groups 1 and 2 (therapeutic group 3).

The second workshop, which involved validating the content of the first version of the Instructive, included 17 participants. The experts assessed the Instructive as valid for use by health teams in the SUS and suggested changes for its improvement.

Suggestions for changes regarding the theoretical frameworks and tools were as follows: supported self-care (discussions on autonomy); transtheoretical model (including questions to assess self-efficacy); cognitive-behavioral therapy (clarifying the psychologist’s strategic role, emphasizing the availability of materials and workshop scripts for the entire health team); therapeutic and motivational groups (defining concepts succinctly, to enhance the contribution of the material to group stratification in the service routine); and workshops (providing guidance on adapting the scripts, when necessary, including incentives and flexibilities to enhance user participation, as well as tailoring to user’s profile, such as age, gender and literacy level, for example). Although not included among the theoretical frameworks and tools adopted as reference in the analysis, a suggestion was identified to incorporate more updated evaluation frameworks, including dimensions of structure, process and performance.

Regarding the care flow, the experts presented suggestions for the four group modalities (motivational, and therapeutic groups 1, 2 and 3), systematized around the following themes: activities, resources, users, professionals and services (details in Table 3).

**Table 3:** Description of the experts’ suggestions from the validation workshop for the motivational group and therapeutic groups

Themes	Motivational group	Therapeutic group 1	Group therapeutic 2	Group therapeutic 3
Activity	- Modify written material - Review duration, sequence and number of meetings - Review scripts - Adapt scripts	- Review the order and topics of the meetings - Include the creation of postcards by users	- Review interval and number of meetings - Review the order and topics of the meetings - Define and standardize the theoretical framework	- Apply exclusively in primary health care - Review the theme and order of meetings
Resource	- Adapt materials to local context (cost and availability) - Adapt to the availability of a multidisciplinary team	- Incorporate communication and information technologies, such as SMS or mobile apps	- Develop strategies for obtaining materials - Develop strategies to identify users’ access profile to information and communication technologies enhance motivation, adherence and integration	- Adapt materials to local context (cost, availability and planning)
User	- Address low adherence rates among male and older users	- Adapt to the level of education and health literacy	- Consider greater demand, as the population in therapeutic group 2 is the most frequent in health centers	- Tailor to meet the expectations and needs of users aiming for weight maintenance or weight regain.
Professional	-	- Assess applicability, based on performance, type and composition of the team - Include the need for planning, clarification and task distribution	- Assess the different levels of expertise among professionals regarding the topic - Integrate strategies for transitioning users to remote activities - Highlight the need for collaborative interprofessional practice.	- Align with collaborative and interprofessional practice and mentoring processes - Define referral and counter-referral flows between primary health care and specialized care - Clarify the facilitator’s role
Service	-	-	- Consider procedures not incorporated into the Unified Health System - Assess the need for a more organized health care network	-

The published version incorporated the problem-posing methodology and food and nutrition education to stimulate a transdisciplinary, intersectoral and multi-professional practice that fosters reflection and autonomy; cognitive-behavioral therapy and the transtheoretical model, aiming to enhance treatment effectiveness and adherence; and the supported self-care tool, recognizing the central role of the user and the team’s shared responsibility. In turn, the workshop was presented as the main educational strategy to support the therapeutic and motivational groups. The diversity of groups, considering the severity of the condition, was used as a tool to enhance the collective approach.

The suggestions for the motivational group were accepted in order to enable users, who lacked the time or desire to participate in groups, to recognize obesity as a problem, particularly those in pre-contemplation, contemplation (regardless of self-efficacy), and preparation with low self-efficacy. As for therapeutic group 1, the recommendations were also accepted, aiming to support the discussion of weight reduction strategies among users in preparation with high self-efficacy or in the action or maintenance stages without indication for surgical treatment or, even, among users with weight regain after surgical treatment. The suggestion for users to create postcards was not accepted, given the objective of this tool to increase adherence to subsequent group activities Instead, differentiation in the postcards was made based on participation or non-participation in the previous meeting.

The published version of the Instructive also incorporated suggestions for therapeutic group 2, supporting the discussion of weight reduction strategies among users in preparation with high self-efficacy or in the action or maintenance stages with indication for surgical treatment of obesity. With regard to therapeutic group 3, the suggestions for the inclusion of topics were accepted, as they contributed to enhancing the users’ confidence.

Different modalities of individual care and the singular therapeutic project were also included in the Strategy for Person with Obesity Care in the SUS, according to the level of care (PHC and SC), aiming to promote comprehensive and longitudinal care. The two pillars of the transtheoretical model (stages of change and self-efficacy) were maintained in the Strategy as key drivers of the flow, along with readiness to participate in groups and the indication for surgical treatment of obesity. The practice of physical exercise and/or physical activity with professional monitoring was incorporated transversally into the Strategy, as well as the longitudinal follow-up through the Family Health Strategy or similar programs.

## Discussion

In this study, the methodological pathway of developing and validating an instructional material was evaluated based on the perceptions and contributions of specialists, in addition to the objectives and frameworks adopted by the organizing team and the General Coordination of Food and Nutrition of the Ministry of Health. At each stage of the development of the Instructive, the experts’ insights were considered, aiming to obtain material with the highest quality and suitability. Thus, it was possible to adapt the recommendations of the *Dietary Guidelines for the Brazilian Population* for the management of obesity, as recommended by the material itself.

During the expert workshop, perceptions were identified regarding aspects related to the theoretical framework and the tool, the health service, the professional and the user. These perceptions were essential for defining which theoretical frameworks and theories would be included in the final summary, guiding the development of the first version of the Instructive and recognizing the need to develop distinct materials for collective and individual approaches. The problem-posing methodology was maintained as a cross-cutting approach, due to its potential to stimulate self-awareness and participation in the care plan. However, in PHC, lectures and expository classes are still frequently used, with less emphasis on problem-posing approaches in educational activities (26). This indicates the need to train professionals in methods, such as problem-posing approach, that promotes the autonomy and empowerment of the participants. From Paulo Freire’s perspective, a liberating education practice considers the learner’s curiosity and knowledge, fostering a horizontal and empowering education that enables individuals to manage their own lives (27). In line with this perspective, the Instructive contributes to directing processes for professional training within the SUS, adopting the perspective of supported self-care and team co-responsibility as key drivers of care.

For the proposed nationwide use of the Instructive, experts emphasized the need to include a recommendation for adapting the workshop scripts according to the characteristics of the teams and available resources. This suggestion was accepted to enable its use in different settings with varying structure and work process in health services. The diversity of the territories was highlighted in a study conducted to describe the adequacy of structure and work processes in the management of obesity, based on data from the national program for improving access and quality (2013-2014), which revealed significant regional variations. Only 26.6% of units had adequate access to obesity management, with the lowest prevalence in the North macro-region. The quality of care was compromised by the lack of adequate infrastructure for diagnosis, evaluation and monitoring (22). Evidence suggests that training processes should be accompanied by the guarantee of infrastructure for the care of people with obesity in PHC, including the availability of equipment in good working conditions (28).

Healthcare professionals working in PHC and SC across all macro-regions of Brazil found other barriers related to the work process, such as low adherence to treatment and the presence of comorbidities. In turn, structural barriers included high demand for emergency and individual care, and a lack of materials and professional training activities (23), which directed the development of the Instructive. Difficulty in adhering to treatment was also highlighted in an analysis of clinical guidelines from different countries revealing that individuals with obesity tend to seek healthcare services only when complications arise (29). Given this scenario, by adopting the stages of change and self-efficacy as key drivers of the Strategy for Person with Obesity Care in the SUS, the Instructive show greater potential to promote adherence to care activities.

The Strategy for Person with Obesity Care in the SUS encompasses care flows according to the level of care (PHC and SC), including collective and individual care modalities to enable comprehensive and longitudinal care for people with obesity. An analysis of the organization of care for people with overweight and obesity in a Brazilian municipality identified weaknesses in comprehensive health care, particularly difficulties in defining the role of each professional (30). These findings underscore the need for investments to improve the quality of health services and care for people with obesity, highlighting a gap that the Instructive can address. Furthermore, considering the challenges of interprofessional and collaborative practice (29,30), the Educational Activities Workbook (19), which accompanies the Instructive, specifies professional categories to conduct the workshops and the level of execution difficulty (16).

Other challenges include updating healthcare professionals in the face of constant production of evidence and information. Therefore, it is essential to facilitate access to instructional materials and promote continuing education in health (30). A qualitative study with public health physicians in an African country identified professional responsibility, personal interest and the need for learning as motivating factors for participating in continuing education activities. On the other hand, lack of relevance to clinical practice, the cost of participation, absence of reward and lack of recognition for staying updated were identified as demotivating factors (31). Thus, while the strengths and methodological rigor of the Instructive were emphasized, its implementation requires coordinated efforts for dissemination within the SUS and professional training across different territories. It is noteworthy that initiatives have already been undertaken to coordinate with professional councils to incorporate it into academic training and continuing education of professionals, along with the availability of free self-instructional courses. In this context, the scale for assessing professionals’ confidence in conducting groups based on the Instructive (32) may help to monitor its contributions to the care for people with obesity within the SUS.

Some positive aspects and limitations of this study should be noted. The content of the scripts was not deeply analyzed, as the study focused on gather evidence on the development and validation of the Instructive, evaluating cross-cutting approaches and theoretical frameworks underlying the proposed educational activities.

As strengths, the inclusion of specialists with different backgrounds and expertise is highlighted as a strategy to develop a material aligned with scientific evidence while also addressing territorial diversity, enhancing its feasibility. The use of a qualitative approach allowed for identifying specialists’ suggestions during face-to-face workshops. Furthermore, it is highlighted that publicizing this development and validation process may contribute to the creation of other instructional materials grounded in scientific evidence and feasible for use by healthcare professionals.

The Instructive were developed following the steps of scientific research, with potential implications for professional training and adoption of practices that promote autonomy, recognizing the central role of users and the co-responsibility of the team. Thus, the potential of the Strategy for Person with Obesity Care in the SUS demonstrates potential for organizing care within the health network, contributing to improving care in light of the growing prevalence of obesity. However, it is also pertinent to emphasize the need for evidence regarding the effectiveness of nutritional interventions based on the Instructive for obesity management, which demands future studies.
