# Supplementary material for: Qualitative methodological pathway for developing the Instructive for the Obesity Management in the SUS, 2018-2020
Source: Epidemiol Serv Saude. 2025 Apr 14;34:e20240365. doi: 10.1590/S2237-96222025v34e20240365.en (PMC11998903; doi:10.1590/S2237-96222025v34e20240365.en)
Supplement: Translation

# Translation: Percurso metodológico qualitativo para elaboração de *Instrutivo de Manejo da Obesidade no SUS*, 2018-2020

**DOI:** 10.1590/S2237-96222025v34e20240365.pt

**Authors:** Thanise Sabrina Souza Santos, Larissa Morelli Ferraz Guimarães, Patrícia Pinheiro de Freitas, Suellen Fabiane Campos, Clareci Silva Cardoso, Natacha Toral, Aline Cristine Souza Lopes

**Affiliations:** 1 Universidade de São Paulo, Núcleo de Pesquisas Epidemiológicas em Nutrição e Saúde, São Paulo, SP, Brasil; 2 Universidade Federal de Minas Gerais, Departamento de Nutrição, Belo Horizonte, MG, Brasil; 3 Universidade Federal dos Vales do Jequitinhonha e Mucuri, Departamento de Nutrição, Diamantina, MG, Brasil; 4 Prefeitura Municipal de Belo Horizonte, Secretaria Municipal de Saúde de Belo Horizonte, Belo Horizonte, MG, Brasil; 5 Universidade Federal de São João Del-Rei, Programa Pós-Graduação em Ciências da Saúde, Divinópolis, MG, Brasil; 6 Universidade de Brasília, Departamento de Nutrição, Brasília, DF, Brasil

**Keywords:** Manejo da Obesidade, Pessoal de Saúde, Educação Continuada, Sistema Único de Saúde, Estudo de Validação

## Abstract

**Objetivo:**

Avaliar o percurso metodológico de desenvolvimento e validação de Instrutivo para Manejo da Obesidade no Sistema Único de Saúde (SUS), com foco nas ações de alimentação e nutrição, a partir de adaptação do *Guia Alimentar para a População Brasileira*.

**Métodos:**

O desenvolvimento do *Instrutivo de Abordagem Coletiva para Manejo da Obesidade no SUS* incluiu avaliação da proposta de sumário, em uma oficina presencial de experts, e análise da primeira versão em uma oficina presencial de validação. Em 2018 e 2019, nas oficinas, realizou-se a dinâmica do carrossel para registrar as percepções de especialistas, atuantes na gestão, academia e serviços de saúde do SUS. Os materiais produzidos nas oficinas foram avaliados por análise de conteúdo exploratória por duas autoras, de forma independente, assumindo referenciais teóricos e teorias como categorias a priori. As divergências observadas foram discutidas até a obtenção de consenso.

**Resultados:**

A primeira oficina incluiu 21 experts e foi essencial para a decisão de desenvolver um material exclusivo para abordagem coletiva e definir os referenciais teóricos e teorias incluídos no Instrutivo. A segunda oficina incluiu 17 experts e contribuiu para a resolução de conceitos, discussões e dúvidas, e consolidação do fluxo de atenção. A versão publicada do Instrutivo abrangeu a metodologia problematizadora de forma transversal e o modelo transteórico como referencial teórico base para potencializar a efetividade e adesão ao tratamento.

**Conclusão:**

O desenvolvimento e a validação do Instrutivo seguiram o percurso de uma pesquisa científica, colaborando com a produção de materiais instrutivos derivados do Guia.

## Introdução

A obesidade é um desafio à saúde pública pelo seu impacto na saúde, qualidade de vida e economia. Análises das tendências mundiais de 1990 a 2022 demonstraram um aumento da prevalência na maioria dos países (1), com projeções de que, em 2034, 54% da população mundial esteja com obesidade (2).

No Brasil, as prevalências de obesidade ratificam a tendência mundial de crescimento, variando de 12% a 24% entre 2006 e 2023, entre adultos (3), podendo alcançar 30% em 2030 (4). Esse cenário epidemiológico reverbera a interação complexa entre os múltiplos determinantes da obesidade (5), com destaque para as mudanças nos padrões alimentares, como o maior consumo de alimentos ultraprocessados (6), que tem sido associado com a obesidade (7,8).

Diante desse contexto, em 2014, foi publicada a segunda edição do *Guia Alimentar para a População Brasileira*, baseada em um novo paradigma da ciência da nutrição, considerando os alimentos e suas combinações, e as dimensões culturais e sociais da alimentação (9). Desde a sua publicação, vários materiais foram publicados visando apoiar a qualificação das ações de promoção da alimentação adequada e saudável (10-16) e a resolutividade do manejo da obesidade no Sistema Único de Saúde (SUS), em concordância com a necessidade de formação profissional (17).

Como um dos desdobramentos do Guia, em 2021, o Ministério da Saúde, em parceria com a universidade, publicou o *Instrutivo de Abordagem Coletiva para Manejo da Obesidade no SUS*, que objetiva apoiar os profissionais de saúde no tratamento de pessoas com obesidade (18). O Instrutivo apresenta a Estratégia para o Cuidado da Pessoa com Obesidade no SUS como proposta para organização do cuidado da obesidade na atenção primária à saúde (APS) e na atenção especializada (AE), e é acompanhado do Caderno de Atividades Educativas, que inclui roteiros de atividades presenciais (oficinas e ações no ambiente) e não presenciais (19).

Apesar de o Instrutivo ter sido recentemente publicado pelo Ministério da Saúde, o seu processo de desenvolvimento ainda não foi cientificamente publicizado. Ao compartilhar o rigor metodológico adotado em sua elaboração, busca-se fortalecer o seu uso na educação permanente e orientação das práticas de cuidado no SUS, bem como contribuir para a produção de materiais instrutivos que auxiliem no manejo de outras condições crônicas. Dessa forma, este artigo objetivou avaliar o percurso metodológico de desenvolvimento e validação do Instrutivo, com foco nas ações de alimentação e nutrição, a partir de adaptação do *Guia Alimentar para a População Brasileira*.

## Métodos

### Contexto

A construção do Instrutivo foi realizada em nove etapas, de 2018 a 2020 (Quadro 1). Neste estudo qualitativo, foram descritas as quatro últimas etapas, por não terem ainda sido publicizadas e serem consideradas cruciais para viabilizar a produção de um material que preenchesse lacunas existentes na orientação do manejo da obesidade e abrangesse novas metodologias de cuidado. Ressalta-se que as demais etapas já foram publicizadas (20-23).

**Quadro 1:** Descrição das etapas de construção do *Instrutivo de Abordagem Coletiva para Manejo da Obesidade no Sistema Único de Saúde*

Etapa	Participantes	Método	Resultado esperado
1. Revisão sistemática	Equipe organizadora	Revisão sistemática da literatura	Reunir evidências científicas para elaboração do Instrutivo
2. Revisão de teorias e ferramentas para o manejo da obesidade	Equipe organizadora	Pré-seleção e discussão de teorias e ferramentas aplicadas no cuidado da pessoa com obesidade	Selecionar teorias e ferramentas utilizadas na atenção primária à saúde para discussão na oficina
3. Diagnóstico da estrutura e processo do manejo da obesidade na atenção primária à saúde	Equipe organizadora	Avaliação de indicadores do programa nacional de melhoria do acesso e da qualidade da Atenção Básica	Conhecer o nível de adequação da atenção primária à saúde para manejo da obesidade
4. Pesquisa com profissionais da atenção primária à saúde e atenção especializada	Equipe organizadora	Pesquisa online	Identificar principais barreiras para manejo da obesidade
5. Proposição do conteúdo do Instrutivo	- Equipe organizadora - Coordenação-Geral de Alimentação e Nutrição do Ministério da Saúde	Construção coletiva	Propor sumário do Instrutivo a ser discutido na oficina
6. Oficina de experts	- Equipe organizadora - Especialistas	Dinâmica do carrossel	Validar proposta de sumário
7. Elaboração do Instrutivo	- Equipe organizadora - Coordenação-Geral de Alimentação e Nutrição do Ministério da Saúde	Revisão das contribuições	Construir primeira versão do Instrutivo
8. Oficina de validação	- Equipe organizadora - Especialistas	Dinâmica do carrossel	Validar primeira versão do Instrutivo
9. Elaboração da versão final do Instrutivo	- Equipe organizadora - Coordenação-Geral de Alimentação e Nutrição do Ministério da Saúde	Revisão das contribuições	Disponibilizar a versão final do Instrutivo para editoração do Ministério da Saúde

### Características dos pesquisadores

A equipe organizadora da elaboração do Instrutivo incluiu profissionais das áreas de nutrição, educação, psicologia e educação física, com atuação em gestão, academia e serviços de saúde do SUS. A construção foi realizada em conjunto com a Coordenação-Geral de Alimentação e Nutrição do Departamento de Prevenção e Promoção da Saúde da Secretaria de Atenção Primária à Saúde do Ministério da Saúde. 

### Instrumento de coleta de dados: oficina de experts

Para apreciação do sumário proposto pela equipe organizadora e o Ministério da Saúde, conduziu-se oficina de experts em agosto de 2018, em formato presencial, com duração de três horas. Foram convidados 26 profissionais de saúde da APS e da AE; gestores municipais, estaduais e federais de saúde; e pesquisadores da temática de obesidade no SUS. Para promover a imersão no referencial teórico, os especialistas receberam previamente o sumário, considerando as evidências científicas reunidas nas etapas anteriores e um compilado de teorias e ferramentas para manejo da obesidade identificadas na etapa número 2 (Quadro 1): abordagens transversais (educação alimentar e nutricional e aconselhamento nutricional); referenciais teóricos (entrevista motivacional, modelo transteórico, terapia cognitivo-comportamental, metodologia problematizadora, e autocuidado apoiado); e ferramentas utilizadas na APS em abordagens individuais (atendimento compartilhado domiciliar, atendimento compartilhado individual, atendimento individual específico e projeto terapêutico singular), familiares (Problem, Roles, Affect, Communication, Time, Illness, Copying, Ecology - P.R.A.C.T.I.C.E. / ciclo de vida / ecomapa) e coletivas (grupos terapêuticos e motivacionais, e oficinas). Esse compilado de teorias e ferramentas foi publicado posteriormente com a denominação de material teórico para suporte ao manejo da obesidade no SUS. (21).

Na oficina de experts, desenvolveu-se a dinâmica do carrossel, quando todos os participantes percorreram as diferentes estações para discutir a proposta de sumário e as teorias e ferramentas pré-selecionadas, acompanhados de integrante da equipe organizadora. Em cada estação, os especialistas avaliaram os pontos positivos e negativos de cada teoria ou ferramenta, bem como sua aplicação no cuidado coletivo e individual de pessoas com obesidade. 

Na perspectiva do modelo de atenção às condições crônicas (24), os especialistas elencaram os referenciais teóricos e as ferramentas para o cuidado e o nível de atenção à saúde (APS ou AE), e o tipo de abordagem a ser empregada (coletiva ou individual), considerando a estratificação da obesidade segundo o índice de massa corporal e a presença de comorbidades (24). Por fim, os especialistas apresentaram sugestões para o sumário final. Posteriormente, a equipe organizadora propôs uma versão consolidada do sumário, integrando as sugestões da oficina. Esse sumário guiou a elaboração da primeira versão do Instrutivo.

### Instrumento de coleta de dados: oficina de validação

No mês de agosto de 2019, em uma segunda oficina, realizou-se a validação do conteúdo do Instrutivo. Também desenvolvida em formato presencial, teve duração de oito horas, e os especialistas foram convidados a avaliarem o conteúdo da primeira versão do material. Os participantes receberam previamente o material para leitura e observações, as quais foram registradas em um instrumento on-line.

Na oficina, foi realizada novamente a dinâmica do carrossel, visando avaliar a adequação dos capítulos do Instrutivo. Em cada estação, foram coletadas as impressões sobre a abrangência do conteúdo e a indicação de conteúdo excessivo ou ausente. Os especialistas circularam entre as estações predefinidas por capítulo com o apoio de um integrante da equipe organizadora. Adicionalmente, avaliaram as propostas terapêuticas, considerando sua aplicabilidade, alinhamento com o SUS, uso de tecnologias, factibilidade, sequência e coerência das atividades apresentadas; e sugestões para a sua maior efetividade.

As considerações apresentadas na oficina foram compiladas pela equipe organizadora e debatidas com a Coordenação-Geral de Alimentação e Nutrição do Ministério da Saúde, e, quando pertinentes, foram incluídas no material. 

### Processamento e análise dos dados

Os produtos das oficinas dos experts e de validação foram avaliados pela análise de conteúdo exploratória (25), para se identificarem as percepções e sugestões dos especialistas e as mudanças que foram incorporadas na versão compilada do sumário do Instrutivo. As teorias e ferramentas para o manejo da obesidade foram consideradas como categorias a priori na análise de conteúdo exploratória, sendo utilizadas como referência na identificação das percepções e sugestões, com sistematização das mudanças e manutenção do conteúdo no sumário e na primeira versão do Instrutivo. 

A análise de conteúdo exploratória foi realizada em planilhas on-line organizadas e compartilhadas entre duas autoras, para viabilizar uma avaliação independente das percepções e sugestões. Todas as divergências observadas foram discutidas até a obtenção do consenso. Para apresentação dos resultados, as percepções e sugestões dos especialistas foram agrupadas em categorias a posteriori, conforme semelhança do conteúdo.

## Resultados

A oficina de experts contou com 21 especialistas, cujas percepções estão resumidas no Quadro 2. Foram identificadas percepções relacionadas ao referencial teórico e à ferramenta (aplicabilidade no cuidado integral da obesidade, viabilizando melhor compreensão dos seus determinantes, porém demanda qualificação); ao serviço de saúde (necessidade de inserir processos de qualificação e matriciamento na rotina, ampliar a equipe de profissionais, reconhecer o território); ao profissional (necessidade de qualificação para uso do método e da prática interprofissional colaborativa); e ao usuário (promoção da adesão, do autoconhecimento e construção de habilidades) (Figura 1).

**Quadro 2:** Síntese das percepções dos especialistas sobre cada abordagem transversal e referencial teórico

Abordagens transversais	
Aconselhamento nutricional	- Prática transversal e multiprofissional, na qual o usuário participa da definição de metas com a equipe de saúde - Requer empatia profissional para evitar a prática verticalizada
Educação alimentar e nutricional	- Útil para auxiliar a equipe na organização do processo de trabalho - Apresenta risco de ser pouco centrada no usuário, se os profissionais a desconhecem
Metodologia problematizadora	- Instrumento robusto de educação em saúde, com potencial para estimular os usuários para o autoconhecimento e a participação no plano de cuidado - Desconhecida por muitos profissionais
Autocuidado apoiado	- Promotora da motivação e autonomia dos usuários - Necessário que os profissionais de saúde identifiquem adequação do seu uso ao perfil dos usuários - Requer materiais de suporte disponíveis no serviço de saúde para promover o seu uso pela equipe
Referenciais teóricos	
Modelo transteórico	- Necessidade de treinamento da equipe, apesar de já ser usado nos serviços de saúde - Pode ser aplicado por qualquer categoria profissional na triagem dos grupos terapêuticos - Maior demanda de tempo e número de profissionais
Entrevista motivacional	- Necessidade de treinamento da equipe, bem como de determinado perfil do profissional para aplicação - Maior demanda de tempo e número de profissionais - Adequada para triagem para grupos terapêuticos, inclusive para manejo da obesidade - Possibilita escuta qualificada e direcionamento adequado do plano de cuidado
Terapia cognitivo-comportamental	- Permite um cuidado interprofissional - Usada em protocolos para condições crônicas - Demanda tempo e treinamento da equipe - Pode requerer participação do profissional de psicologia
Projeto terapêutico singular	- Usada em casos mais complexos - Limitações: dificuldade de acompanhamento longitudinal e não participação do usuário na construção do plano de cuidado
Referenciais teóricos	
Atendimento compartilhado individual	- Possibilita soma de saberes coletivos, sendo estratégico no matriciamento e para superar hierarquia de conhecimentos - Requer tempo e disponibilidade para alinhamento dos profissionais
Atendimento individual específico	- Favorece adesão ao tratamento - Oportuniza aprofundamento em questões delicadas, agregando competências específicas ao cuidado - A integração com a abordagem coletiva pode ser uma dificuldade
Atendimento compartilhado domiciliar	- Possibilita identificar as vulnerabilidades do domicílio e especificidades do núcleo familiar - Viabiliza a presença dos agentes comunitários de saúde junto ao profissional com nível superior
P.R.A.C.T.I.C.E^a^, ciclo de vida e ecomapa	- Requer tempo, considerando-se que a interface com o prontuário eletrônico é incipiente e existe normatização para inserção das ferramentas no processo de trabalho - Escassez de evidências científicas que sustentem a recomendação do seu uso - Apresentam potencial para melhor compreensão dos determinantes da obesidade, com promoção do reconhecimento das diferentes configurações familiares e da estruturação do projeto terapêutico singular
Grupos terapêuticos e motivacionais	- Úteis para organização do cuidado e mais efetivos, por reunirem usuários que compartilham problemas em comum, favorecendo a troca de experiências - Necessidade de os profissionais de saúde terem habilidades para condução/mediação - A participação dos usuários depende bastante do nível de motivação para mudança de comportamento e do reconhecimento da obesidade como um problema
Oficinas	- O processo de planejamento e condução pode reduzir a efetividade - Permitem aos profissionais desenvolverem habilidades terapêuticas e motivacionais - Necessidade de formação profissional para que a equipe oferte oficinas no seu maior potencial

**Figura 1:** Percepções dos experts distribuídas segundo relação com referencial teórico ou ferramenta, serviço, profissional e usuário

A partir da discussão das ferramentas e de sua aplicabilidade no cotidiano de trabalho, os especialistas recomendaram que houvesse um Instrutivo focado na abordagem coletiva e outro na abordagem individual. Dessa forma, entre os referenciais teóricos e teorias analisados na oficina de experts, no sumário final foram omitidos: aconselhamento nutricional, projeto terapêutico singular, atendimento compartilhado individual, atendimento compartilhado domiciliar, atendimento individual específico, e P.R.A.C.T.I.C.E. / ciclo de vida / ecomapa. 

Em relação ao fluxo de atenção, os especialistas sugeriram que a definição da abordagem coletiva deveria considerar a prontidão de mudança para redução do peso corporal, a gravidade da obesidade e a presença de comorbidades, especialmente diabetes. Para isso, a avaliação inicial deveria incluir a identificação dos estágios de mudança, do grau de obesidade e comorbidades existentes, assumindo o modelo transteórico e a terapia cognitivo-comportamental, baseados na abordagem problematizadora, como referenciais teóricos para orientação do cuidado centrado na pessoa e definição dos grupos terapêuticos. Dessa forma, o fluxo de atenção, na primeira versão do Instrutivo, deveria se iniciar a partir dos estágios de mudança e da autoeficácia, constructos do modelo transteórico, e considerar a disponibilidade para participar de grupos e estratificação da condição de saúde, incluindo a indicação para o tratamento cirúrgico da obesidade. O fluxo incluía grupos terapêuticos para: A) Pessoas nos estágios de pré-contemplação, contemplação e preparação com baixa autoeficácia; e/ou sem disponibilidade para grupos (grupo motivacional); B) Pessoas nos estágios de preparação e elevada autoeficácia, ação ou manutenção sem indicação de tratamento cirúrgico (grupo terapêutico 1); com indicação de tratamento cirúrgico (grupo terapêutico 2); e em acompanhamento pós-cirúrgico ou com sucesso nos grupos terapêuticos 1 e 2 (grupo terapêutico 3).

A segunda oficina, que envolveu a validação do conteúdo da primeira versão do Instrutivo, incluiu 17 participantes. Os especialistas avaliaram o Instrutivo como válido para ser utilizado pelas equipes de saúde no SUS e sugeriram mudanças para o seu aperfeiçoamento. 

As sugestões de mudança em relação aos referenciais teóricos e às ferramentas foram as seguintes: autocuidado apoiado (discutir sobre autonomia); modelo transteórico (incluir questões para avaliação da autoeficácia); terapia cognitivo-comportamental (esclarecer o papel estratégico do psicólogo, reforçando sobre a disponibilidade de materiais e roteiros de oficinas para toda a equipe de saúde); grupos terapêuticos e motivacionais (definir os conceitos de forma sucinta, para potencializar a contribuição do material para estratificação de grupos na rotina do serviço); e oficinas (orientar para adaptar os roteiros, quando necessário, incluindo-se incentivos e flexibilizações para potencializar a participação dos usuários, bem como adequação ao perfil dos usuários quanto à idade, gênero e nível de alfabetização, por exemplo). Apesar de não constar entre as teorias e ferramentas assumidas como referência no corpo da análise, foi identificada sugestão de inserir marcos teóricos de avaliação mais atualizados, incluindo as dimensões de estrutura, processo e desempenho.

Em relação ao fluxo de atenção, os especialistas apresentaram sugestões para as quatro modalidades de grupo (motivacional, e terapêuticos 1, 2 e 3), sistematizadas em torno dos seguintes temas: atividade, recurso, usuário, profissional e serviço (detalhes no Quadro 3).

**Quadro 3:** Descrição das sugestões dos especialistas na oficina de validação para o grupo motivacional e os grupos terapêuticos

Temas	Grupo motivacional	Grupo terapêutico 1	Grupo terapêutico 2	Grupo terapêutico 3
Atividade	- Alterar escrita - Revisar duração, ordem e quantidade de encontros - Revisar roteiros - Adaptar roteiros	- Revisar ordem e temas dos encontros - Incluir elaboração dos cartões postais pelos usuários	- Revisar intervalo e número dos encontros - Revisar ordem e temas dos encontros - Definir e padronizar o referencial teórico	- Aplicar apenas na atenção primária à saúde - Revisar temática e ordem dos encontros
Recurso	- Adequar materiais à realidade local (custo e disponibilidade) - Adequar à disponibilidade de equipe multiprofissional	- Incluir tecnologias de comunicação e informação, como mensagem via SMS ou aplicativo	- Incluir estratégias para obtenção dos materiais - Incluir estratégias para identificar perfil de acesso dos usuários às tecnologias de informação e comunicação para maior motivação, adesão e integração	- Adequar materiais à realidade local (custo, disponibilidade e planejamento)
Usuário	- Atentar para adesão fragilizada de usuários do sexo masculino e idosos	- Adequar ao grau de escolaridade e letramento em saúde	- Ponderar a maior demanda, uma vez que o público do grupo terapêutico 2 é o mais frequente nas unidades de saúde	- Adequar às expectativas e necessidades para atender perfis de manutenção ou reganho de peso
Profissional	-	- Avaliar aplicabilidade, dependendo do desempenho, tipo e composição da equipe - Incluir necessidade de planejamento, esclarecimento e distribuição das funções	- Avaliar quanto aos diferentes níveis de domínio do tema pelos profissionais - Incluir estratégias para retorno dos usuários em atividades não presenciais - Atentar para demanda de prática interprofissional colaborativa	- Alinhar à prática colaborativa e interprofissional e ao matriciamento - Indicar fluxos de referência e contrarreferência entre atenção primária à saúde e atenção especializada - Esclarecer papel do facilitador
Serviço	-	-	- Ponderar sobre procedimento não incorporado no Sistema Único de Saúde - Avaliar necessidade de rede de atenção à saúde mais organizada	-

A versão publicada incorporou a metodologia problematizadora e a educação alimentar e nutricional, para estimular uma prática transdisciplinar, intersetorial e multiprofissional geradora de reflexão e autonomia; a terapia cognitivo-comportamental e o modelo transteórico, visando potencializar a efetividade e a adesão ao tratamento; e a ferramenta do autocuidado apoiado, reconhecendo o papel central do usuário e a corresponsabilidade da equipe. Por sua vez, a oficina foi apresentada como a principal estratégia educativa para subsidiar os grupos terapêuticos e o motivacional. A diversidade de grupos, considerando-se a gravidade da condição, foi utilizada como ferramenta visando potencializar a abordagem coletiva.

As sugestões para o grupo motivacional foram acatadas de forma a propiciar o reconhecimento da obesidade como um problema pelos usuários que não tinham tempo ou desejo para participar de grupos, em pré-contemplação, contemplação (independente da autoeficácia) e em preparação com baixa autoeficácia. Em relação ao grupo terapêutico 1, os apontamentos também foram acatados, visando subsidiar a discussão de estratégias para a redução de peso entre usuários em preparação com alta autoeficácia ou nos estágios de ação ou manutenção sem indicação para tratamento cirúrgico ou, ainda, entre usuários com reganho de peso após o tratamento cirúrgico. Não foi acatada somente a sugestão para que os usuários elaborassem os cartões postais, considerando-se o objetivo dessa ferramenta para aumentar a adesão às próximas atividades do grupo. Optou-se por uma diferenciação nos cartões postais, levando-se em conta a participação ou não no encontro anterior.

A versão publicada do Instrutivo também acolheu as sugestões para o grupo terapêutico 2, subsidiando a discussão de estratégias para redução de peso entre usuários em preparação com alta autoeficácia ou nos estágios de ação ou manutenção com indicação para tratamento cirúrgico da obesidade. Em relação ao grupo terapêutico 3, as sugestões para inclusão de temáticas foram acatadas, por contribuírem para ampliação da confiança dos usuários.

Diferentes modalidades de atendimentos individuais e o projeto terapêutico singular também foram incluídos na Estratégia de Cuidado da Pessoa com Obesidade no SUS, segundo nível de atenção (APS e AE), visando favorecer o cuidado integral e longitudinal. Os dois pilares do modelo transteórico (estágios de mudança e autoeficácia) foram mantidos na Estratégia como direcionadores chaves do fluxo, junto com a disponibilidade para participar de grupos e a indicação para tratamento cirúrgico da obesidade. A prática de exercício físico e/ou atividade física com acompanhamento profissional foi inserida de forma transversal na Estratégia, assim como o acompanhamento longitudinal da Estratégia Saúde da Família ou similar.

## Discussão

Neste estudo, avaliou-se o percurso metodológico de desenvolvimento e validação de material instrutivo, a partir das percepções e contribuições de especialistas, em adição aos objetivos e referenciais assumidos pela equipe organizadora e a Coordenação-Geral de Alimentação e Nutrição do Ministério da Saúde. Em cada etapa de construção do Instrutivo, foram considerados os apontamentos dos especialistas, visando obter um material com a máxima qualidade e adequação possível. Dessa forma, foi possível adaptar as recomendações do *Guia Alimentar para a População Brasileira* para o manejo da obesidade, conforme recomendado pelo próprio material. 

Na oficina de experts, foram identificadas percepções em torno de aspectos relacionados ao referencial e à ferramenta, ao serviço de saúde, ao profissional e ao usuário. Tais percepções foram essenciais para definir quais referenciais teóricos e teorias seriam incluídos no sumário final, orientar a elaboração da primeira versão do Instrutivo e reconhecer a necessidade de desenvolver as abordagens coletiva e individual em materiais distintos. A metodologia problematizadora foi mantida como abordagem transversal, por seu potencial para estimular os usuários para o autoconhecimento e a participação no plano de cuidado. Entretanto, ainda se constata na APS o uso de palestras e aulas expositivas e uma menor frequência da problematização nas atividades educativas realizadas (26). Isso denota a necessidade da qualificação dos profissionais para uma abordagem problematizadora e promotora da autonomia e do protagonismo dos participantes. Na perspectiva freiriana, a prática de uma educação libertadora considera a curiosidade e o conhecimento do educando, construindo-se uma educação horizontal e empoderadora que promove a libertação para o gerenciamento de suas vidas (27). Alinhado a essa discussão, o Instrutivo contribui para orientar processos de qualificação de profissionais no SUS, assumindo a perspectiva do autocuidado apoiado e da corresponsabilidade da equipe como potencializadores do cuidado.

Na proposição de uso nacional do Instrutivo, os especialistas ressaltaram a necessidade de incluir a recomendação para adaptar os roteiros das oficinas conforme as características das equipes e recursos disponíveis. A sugestão foi acatada, visando viabilizar o seu uso em diferentes cenários de estrutura e processo de trabalho nos serviços de saúde. A diversidade dos territórios foi apontada em estudo conduzido para descrever a adequação da estrutura e do processo de trabalho no manejo da obesidade, a partir dos dados do programa nacional de melhoria do acesso e da qualidade (2013-2014), que revelou variações regionais significativas. Apenas 26,6% das unidades apresentaram acesso adequado ao manejo da obesidade, com a menor prevalência na macrorregião Norte. A qualidade do cuidado foi comprometida pela falta de estrutura adequada para diagnóstico, avaliação e monitoramento (22). Evidências sugerem que a oferta de processos de qualificação deve ser acompanhada da garantia de infraestrutura para o cuidado da pessoa com obesidade na APS, incluindo a disponibilidade de equipamentos em condições adequadas de uso (28).

Profissionais de saúde com atuação na APS e na AE de todas as macrorregiões do Brasil constataram outras barreiras relacionadas ao processo de trabalho, como baixa adesão ao tratamento e presença de comorbidades. Por seu turno, as barreiras estruturais identificadas foram a alta demanda por atendimento emergencial e individual, e a escassez de materiais e atividades de qualificação profissional (23), sendo esses resultados orientadores do desenvolvimento do Instrutivo. A dificuldade de adesão ao tratamento também foi apontada em análise de diretrizes clínicas de diferentes países, constatando-se que usuários com obesidade tendem a procurar serviços de saúde somente quando vivenciam complicações (29). Diante desse cenário, ao assumir os estágios de mudança e a autoeficácia como direcionadores chaves da Estratégia de Cuidado da Pessoa com Obesidade no SUS, o Instrutivo se mostra com maior potencial para promover a adesão às atividades.

A Estratégia de Cuidado da Pessoa com Obesidade no SUS abarca fluxos segundo o nível de atenção (APS e AE), incluindo modalidades de atendimentos coletivos e individuais para viabilizar a integralidade e longitudinalidade no cuidado da pessoa com obesidade. Análise da organização do cuidado às pessoas com sobrepeso e obesidade em um município brasileiro identificou fragilidades na atenção integral à saúde, com destaque para a dificuldade em definir o papel de cada profissional (30). Tais resultados reforçam a demanda por investimentos para qualificar a oferta de serviços de saúde e o cuidado de pessoas com obesidade, sinalizando a lacuna que o Instrutivo pode preencher. Ademais, frente às dificuldades para uma atuação interprofissional e colaborativa (29,30), o *Caderno de Atividades Educativas* (19), que acompanha o Instrutivo, inclui a indicação de categorias profissionais para condução das oficinas e o nível de dificuldade de execução (16).

Outros desafios incluem a atualização dos profissionais de saúde frente à constante produção de evidências e informações. Nesse sentido, é essencial facilitar o acesso aos materiais instrutivos e promover a educação permanente em saúde (30). Um estudo qualitativo com médicos de saúde pública, em um país do continente africano, identificou a responsabilidade profissional, o interesse pessoal e a necessidade de aprendizagem como fatores motivadores para participação em atividades de educação permanente. Por outro lado, a não relevância para a prática clínica, o custo da participação, a falta de recompensa e nenhum reconhecimento por se manter atualizado foram fatores desmotivadores (31). Assim, ao mesmo tempo que as potencialidades e o rigor metodológico do Instrutivo foram ressaltados, a sua implementação requer esforços coordenados para sua disseminação no SUS e qualificação dos profissionais nos diferentes territórios. Destaca-se que já foram realizadas ações de articulação com os conselhos profissionais, para uso na formação acadêmica e educação permanente dos profissionais, e disponibilizados cursos autoinstrucionais e gratuitos. Nesse sentido, a escala para avaliar a confiança dos profissionais para a realização de grupos a partir do Instrutivo (32) poderá contribuir para monitorar as suas contribuições para o cuidado da pessoa com obesidade no SUS. 

Alguns aspectos positivos e limitações deste estudo devem ser apontados. O conteúdo dos roteiros não foi analisado profundamente, uma vez que o enfoque do estudo foi reunir evidências do desenvolvimento e validação do Instrutivo, avaliando as abordagens transversais e os referenciais teóricos que sustentaram a proposição das atividades educativas. 

Como potencialidades, aponta-se a inclusão de especialistas com diferentes formações e atuações como estratégica para viabilizar a construção de um material alinhado com evidências científicas, mas, também, que contempla as multiplicidades dos territórios, potencializando a sua factibilidade. O uso de uma abordagem qualitativa viabilizou identificar as sugestões dos especialistas nas oficinas presenciais. Ademais, destaca-se que a publicização desse processo de construção e validação poderá contribuir para a produção de outros materiais instrutivos pautados em evidências científicas e factíveis para uso pelos profissionais de saúde. 

O Instrutivo foi desenvolvido seguindo o percurso de uma pesquisa científica, tendo possíveis implicações para a qualificação profissional e adoção de práticas promotoras da autonomia com reconhecimento do papel central dos usuários, mas, também, da corresponsabilização da equipe. Reconhece-se, assim, o potencial da Estratégia para o Cuidado da Pessoa com Obesidade no SUS para a organização do cuidado na rede de atenção à saúde, contribuindo para potencializar a assistência frente à prevalência crescente da obesidade. Mas, também, é oportuno ressaltar a necessidade de reunir evidências quanto à efetividade de intervenções nutricionais baseadas no Instrutivo para o manejo da obesidade, o que demanda estudos futuros.
